# The Mind

**Published:** 1856-05

**Authors:** 


### THE MIND.

That mysterious thing, the God within us, which no eye can see, whose dwelling place none can tell, yet of whose presence the habitable globe gives note, how inexorably does it govern the body, whose instrument it is; how it makes or mars the human form divine; how it blanches the ruddiest cheek; how it dims the lustrous eye; how it bends in a night the stateliest carriage, and in a night frosts over the raven ringlet; in an hour strikes down the strength of manhood, and in a moment can make itself a blank for the balance of the lifetime of its crazed tenement; how important to keep that agent *well;* how heaven-like the skill to minister to a mind diseased. Few persons have an adequate conception of the importance and frequent need of mental medication. "The very trip of your feet along the corridor makes me feel half well again, Doctor," said a Catholic priest to me one day as I entered his cheerless room; as cheerless and cold, too, must be every room where the wife and the child can never come to brighten and to happify.

As any man of good observation is his body's own best physician for ordinary slight ailments, so the mind may be rendered by proper tuitions its safest and most efficient doctor. These tuitions should be early begun; they should commence with the toddling infant of a year, by letting it learn to locomote itself, by giving it an opportunity of trying to get up the very first time it falls on the floor. In a thousand little ways may any parent of good common sense implant a germ—*the habit of self-reliance*—whose subsequent fruitage may be the glory of the nation; *self-reliance*, more priceless than any diadem that ever graced a monarch's brow; a "security" which the "tightest times" only serve to improve; self-reliance which falters in no strait, which pales before no obstacle, which no disaster can paralyze, no calamity appal. "*Rod dot it, I'll try it again*," said a ragged little urchin as he slipped and fell under a heavy piece of timber which he was carrying to his mother one bleak winter's day, and no sooner said than done, and up he jumped, and raising the timber to his shoulder, was soon lost in the crowd as to sight, but not in sound, for some operatic notes about "supper" and "*old Dan Tucker*" showed a cheery heart within him, and that he felt there was gladness at home for him. Who doubts either of two things; that that boy had a noble mother at home, and that if he lives he will be a man of mark in the community about him?

Within a month our city was startled by the sudden and unexpected death of one of the leading members of a mercantile firm who became bankrupt a few days before, originating in the villany of a partner several years ago. He was a man of noble bearing and of a proud spirit, but the outrageous abuse of two or three remorseless creditors, in the presence of his clerks and others, so weighed upon his spirits, that he died within forty-eight hours. For his sensitiveness, we owe him our love and sympathy, and a monument to his memory will we give for the bravery of an eight years' effort to retrieve the losses which another brought upon him, then ran away; but for the last act of his life, the permission of a *broken heart*, figuratively speaking, we hold him accountable to the bar of society, as no man has a right to flee on the occurrence of any financial disaster, for the simple reason that his personal explanations can always lessen the losses of his friends by enabling them the better to gather up the fragments, so no man has a right to run away from himself to take refuge in death by cherishing the remorses of an injured spirit, especially when, as in this case, those remorses arise from a miseducated integrity or a miseducated conscience as to financial matters. It is immaterial what Mrs. Grundy will say, or what the world may think of our conduct, as long as we are conscious of a well-informed mercantile integrity. With that, a man may utterly fail half a dozen times, and stand the higher after each successive failure, as did *Josiah Lawrence* of Cincinnati; and with a proper portion of the self-reliance of the ragged and overburdened boy on the street, such a man will die at last, in the most desirable sense of the word, "A SUCCESSFUL MAN."

